# Detection of spatial frequency in brain-damaged patients: influence of hemispheric asymmetries and hemineglect

**DOI:** 10.3389/fnhum.2013.00092

**Published:** 2013-04-03

**Authors:** Natanael A. dos Santos, Suellen M. Andrade, Bernardino Fernandez Calvo

**Affiliations:** Laboratory of Perception, Neuroscience and Behavior, Department of Psychology, Federal University of ParaíbaJoão Pessoa, Brazil

**Keywords:** hemispheric specialization, spatial frequency, hemineglect, contrast sensitivity, stroke

## Abstract

Hemispheric specialization for spatial frequency processing was investigated by measuring the contrast sensitivity curves of sine-wave gratings in 30 left or right brain-damaged patients using different spatial frequencies compared with healthy participants. The results showed that left brain-damaged patients were selectively impaired in processing high frequencies, whereas right brain-damaged patients were more impaired in the processing low frequencies, regardless of the presence of visuo-spatial neglect. These visual processing results can be interpreted in terms of spatial frequency discrimination, with both hemispheres participating in this process in different ways.

## Introduction

The role of factors related to hemispheric asymmetry in visual information processing has been long considered and debated in studies of perception (Koningsbruggen et al., 2010; Stevens et al., 2012). With regard to the anatomical and physiological aspects of visual processing, parallel groups of visual pathways consist of parvocellular cells that respond better to medium and high luminance patterns and magnocellular cells that respond better to low luminance patterns. These layers are involved in differences in achromatic contrast sensitivity under scotopic and photopic luminance conditions, respectively. These two pathways continue separately to distinct layers of the lateral geniculate nucleus of the primary visual cortex (M pathway for 4Cα layer and P pathway for 4Cβ layer; Pokorny et al., 2003). With regard to the contrast sensitivity of these cells, the parvocellular pathway is involved in the spatial resolution of medium and high spatial frequencies, and the magnocellular pathway responds mainly to low spatial frequencies (Silva et al., 2011).

One controversial hypothesis that is related to spatial frequency detection concerns the fact that high-frequency stimuli are processed faster and more accurately by the left hemisphere (LH). Conversely, the right hemisphere (RH) is involved in low-frequency processing (Keenan et al., 1989; Iidakaa et al., 2004). Nonetheless, no consensus has been reached about the hemispheres' roles in medium spatial frequency processing or how visual neglect could be implicated in this processing (Hellige, 1995; Grabowska and Nowicka, 1996; Karnath and Niemerier, 2002).

Therefore, the aim of the present study was to analyze contrast sensitivity thresholds in patients with LH and RH lesions, with and without hemineglect, making use of low, medium, and high spatial frequencies and comparing their performance with healthy individuals.

## Methods

### Participants

Forty volunteers participated in the study. These volunteers were selected for their accessibility and age (between 40 and 65 years old). The patients were divided into four subgroups, each with 10 participants (five women and five men): (1) 10 healthy volunteers [*M* = 49.5 years, *SD* = 3.83 years; control group (CG)], (2) 10 right brain-damaged patients (*M* = 51.4 years, *SD* = 3.86 years) affected by visuo-spatial neglect (RN+ group), (3) 10 right brain-damaged patients (*M* = 50.5 years, *SD* = 4.52 years) without any sign of visuo-spatial neglect (RN− group), and (4) 10 left brain-damaged patients (*M* = 50.4 years, *SD* = 4.19 years) not affected by visuo-spatial neglect (LN− group).

Upon admission, all of the patients underwent clinical neuropsychological assessment and a standard neurological exam. Neurological assessment was based on the National Institute of Health Stroke Scale (Brott et al., 1989).

The inclusion criteria adopted in the present study included the diagnosis of non-recurring unilateral ischemic stroke, acute stage (occurring at least 1 month after the vascular event), injury to the middle cerebral artery, and normal or corrected-to-normal visual acuity. Data were obtained from medical records and functional magnetic resonance imaging. Participants in the CG were healthy individuals who were accompanying the patients or working in the institution where the experiment was performed. The pathological diagnosis was performed based on the International Statistical Classification of Diseases and Related Health Problems, 10th revision.

The exclusion criteria were hemorrhagic stroke, recurring, extensive cerebral lesion, incapability of completing the interview and assessment because of serious aphasia, psychiatric dysfunction, ocular disease, unconsciousness, or use of drugs that modulate activity of the central nervous system.

In the CG, the Cumulative Illness Research Scale was applied to guarantee the participation of healthy individuals in this group. This scale investigates the presence of 14 disease sets (i.e., cardiac, vascular, hematological, respiratory, ocular, upper and lower gastrointestinal tract, hepatic and pancreatic, renal, genitourinary, musculoskeletal and integumental, neurologic, endocrine-metabolic, breast, and psychiatric), taking into consideration situations in which each set of diseases is absent, mild, moderate, severe, or extremely severe, with scores ranging from 0 to 4, respectively (Fortin et al., 2011). Personal data and lesion site are reported in Table 1.

**Table 1:** **Personal data and lesion site of patients**.

**Subject**	**Group**	**Sex**	**Age**	**Education (years)**	**Lesion site**
**LEFT-HEMISPHERE LESION**					
1	LN−	F	49	5	FT
2	LN−	M	47	6	FP
3	LN−	M	53	5	TP
4	LN−	F	49	5	co
5	LN−	F	57	7	TP
6	LN−	F	46	5	P
7	LN−	M	50	5	FT
8	LN−	M	51	10	FTP
9	LN−	F	45	5	T
10	LN−	M	57	15	FT
**RIGHT-HEMISPHERE LESION**					
11	RN−	F	45	6	FT
12	RN−	F	49	6	P
13	RN−	F	50	5	FT
14	RN−	M	46	5	ic
15	RN−	M	55	5	TP
16	RN−	F	49	8	FTP
17	RN−	F	53	15	TP
18	RN−	M	45	5	FTP
19	RN−	M	56	5	FTP
20	RN−	M	57	5	T
21	RN+	M	57	5	TP
22	RN+	F	55	5	FT
23	RN+	F	45	5	T
24	RN+	M	52	8	FPcr
25	RN+	M	50	5	FTP
26	RN+	F	47	10	TP
27	RN+	M	49	5	FT
28	RN+	F	54	7	FP
29	RN+	F	55	5	FTP
30	RN+	M	50	5	P

F, frontal lobe; P, parietal lobe; T, temporal lobe; co, centro ovalis; cr, corona radiata; ic, internal capsule.

All of the participants were administered the Frontal Assessment Battery (Dubois et al., 2000) and Mini-Mental State Examination (Folstein et al., 1975). Before being recruited for the study, all of the participants were subjected to an extensive neuropsychological evaluation that assessed language (Kaplan et al., 1983), constructional apraxia (Arrigoni and De Renzi, 1964), episodic memory (Spinnler and Tognoni, 1987), and visuo-spatial function (Spreen and Strauss, 1991). Heminegligence was assessed using the Behavioral Inattention Test, a battery of tests that evaluates spatial deficits, including both conventional and behavioral scales (Wilson et al., 1987).

No between-group differences were found in general severity of the neuropsychological consequences of the stroke (Table 2), with the exception of the presence of aphasia in some left brain-damaged patients and presence of visuo-spatial neglect in some right brain-damaged patients.

**Table 2:** **Results obtained at the neuropsychological assessment by the LN−, RN−, and RN+ patients**.

**Test**	**LN−**	**RN−**	**RN+**	***p*-value**
Frontal assessment battery (0–18)	14.7 (2.3)	15.5 (3.1)	12.9 (1.3)	0.09
Mini-mental state examination (0–30)	22.7 (1.2)	23.2 (1.5)	19.7 (2.1)	0.07
Naming test (0–15)	8.8 (4.1)	14.3 (3.3)	14.1 (2.9)	0.04^*^
Constructional praxia (0–11)	7.3 (2.1)	9.6 (2.7)	5.2 (2.1)	0.08
Verbal memory (0–10)	4.7 (1.3)	6.8 (2.4)	5.1 (1.9)	0.09
Street's completion test (0–14)	8.2 (3.4)	10.1 (4.2)	6.8 (3.3)	0.05
BIT-C (0–146)	138.7 (3.2)	140.1 (2.7)	72.1 (2.4)	0.02^*^
BIT-B (0–81)	74.5 (1.1)	73.1 (1.4)	40.6 (2.3)	0.03^*^

* p < 0.05.

The participants were informed about the study protocol and objective of the experiment in compliance with the Declaration of Helsinki. The local Ethics Committee approved the study.

### Equipment and stimuli

The stimuli were set to appear in the center of a 19-inch cathode ray tube video monitor (LG) with 1024 × 768 pixel resolution and a 70 Hz frame rate. Inputs were controlled by a computer through a video board with VGA and DVI connectors. The voltage luminance of the monitor was expanded from 8 to 14 bits using BITS++ (Cambridge Research Systems, Rochester, Kent, England, 2002), allowing the use of visual stimuli with lower contrast gradations. LightScan software, equipped with OptiCAL Photometry (Cambridge Research Systems, Rochester, Kent, England, 2002), was used to measure screen luminance, and gamma correction of the monitor was performed using 48 index values, ranging from 0 to 255 (gamma = 1.8) as a sample. The lowest and highest luminance values of the screen were 0.20 and 80.0 cd/m^2^, respectively (mean luminance = 40.1 cd/m^2^). The room was 2.5 × 2.0 m in size and illuminated by a 20 W fluorescent bulb (Philips). The walls of the room were gray, which allowed for better control of the room lightning conditions during the experiment. A C++ computer program, developed by the responsible lab, was used to run the experiment, generated the stimuli, controlled stimulus presentation, and recorded contrast thresholds.

**Figure 1:**
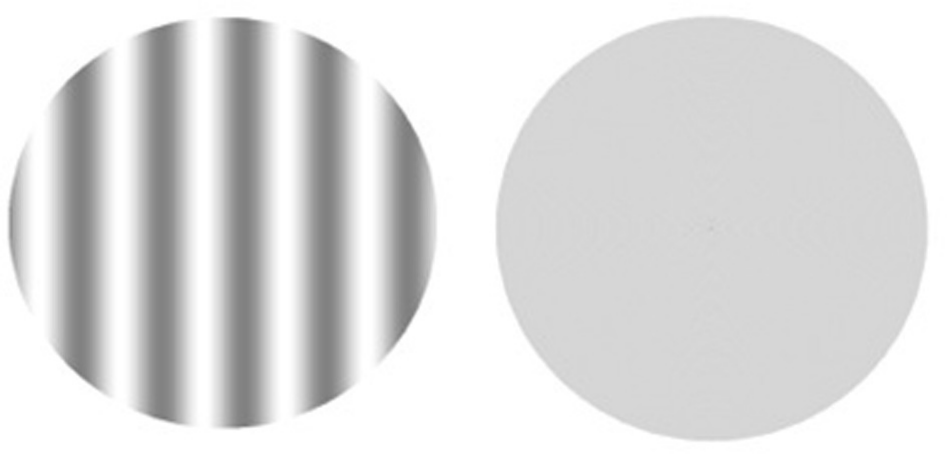
**Example of a pair of stimuli.** The left side shows the 0.25 cpd spatial frequency, and the right side shows the neutral stimulus. The stimuli were originally calibrated to be seen at a distance of 150 cm.

Achromatic and vertical static sine-wave grating stimuli with spatial frequencies of 0.25, 2.0, and 4.0 cycles per degree (cpd) of visual angle were used in the study (Figure 1).

All of the stimuli had a diameter of ~7.2° of visual angle and were designed to be presented in the middle of the monitor at a distance of 150 cm from the observer.

### Procedure

The contrast sensitivity of all participants was estimated using a psychophysical method with forced-choice between two temporal alternatives originally proposed by Wetherill and Levitt (1965). The forced-choice method is based on the probability of consecutive hits by the subject, i.e., about 100 opportunities to choose between two stimuli (test stimulus and neutral stimulus). The test stimulus (sine-wave grating) is perceived by the volunteer 79% of the time.

The procedure for measuring the threshold for each frequency consisted of presenting successive pairs of simple stimuli in which one was the test stimulus, which should be identified by the participant (the first or second stimulus of each par). The order of presentation of stimuli and the frequencies was random and controlled by the program. The criteria used to measure the contrast sensitivity for each spatial frequency was three consecutive hits to decrease 20% of the contrast, and an error to increase contrast by the same percentage (Dos Santos and Andrade, 2012).

A stimuli sequence was presented during each experimental session, starting with a beep, followed immediately by the presentation of the first stimulus for 2 s, then there was 1 s interval between stimuli, followed by the presentation of the second stimulus for 2 s and the volunteer's response. When the volunteer response was correct, it was followed by another beep. The interval between trials was 3 s regardless of the answer (or choice) to be correct or wrong. The beep indicating the beginning of stimuli pair presentation and that indicating the correct choice were different.

All volunteers received the following statement: “pairs of circles will appear on the screen, one after the other. One of them will be totally gray, while the other will contain light and dark stripes. When the circle with stripes appears first, you must press the left mouse button (button 1); when the circle with stripes appears in second place, you should press the right mouse button (button 2).” The task of the volunteer was to always choose the stimulus that contained the spatial frequency. Each session began with the test stimulus contrast at a supra-threshold level, and the experiments began only when the investigator was convinced that the participant understood the directions and responded as instructed. All measurements were performed at a 150 cm distance with binocular vision and natural pupil.

In this type of procedure, the number of presentations of stimuli pairs is variable and depends on the success of the volunteer as well as the number of maximum and minimum or reversal values previously defined. In this study, each experimental session ended automatically after six threshold values (three maximum and three minimum) or six reversals obtained by the participant, which took on average 10–15 min. Each one of the points (or frequencies) of the contrast sensitivity curve was estimated at least twice (two experimental sessions), on different days, for each of the participants.

## Results

The contrast sensitivity threshold was analyzed using a split-plot analysis of variance (ANOVA), with group (C, RN+, RN−, and LN−) as the between-subjects factor and spatial frequency (low, medium, and high) as the within-subjects factor. A significant effect of group was found [*F*_(3, 36)_ = 49.85, *p* < 0.01] but no effect of frequency [*F*_(2, 36)_ = 2.41, *p* < 0.21].

**Figure 2:**
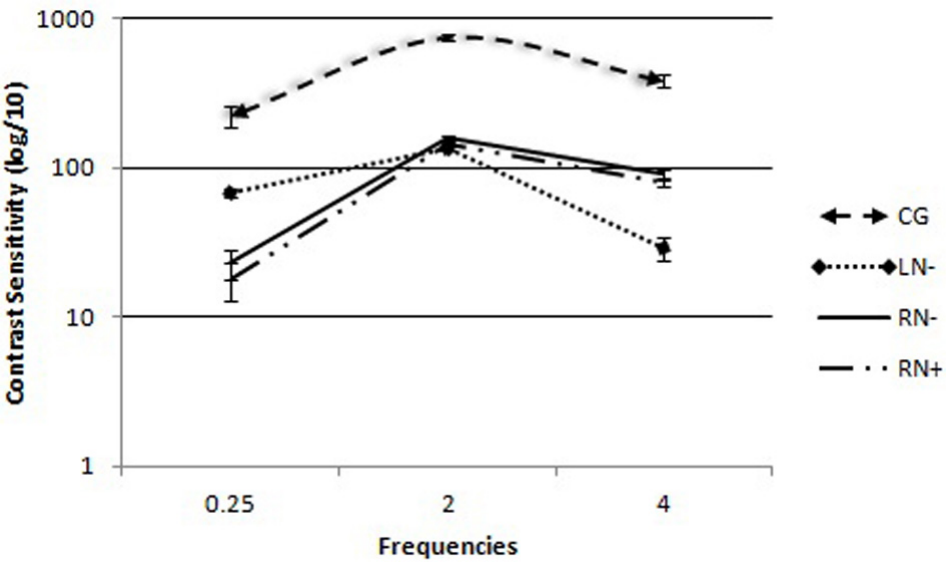
**Contrast sensitivity curves for spatial frequency in each group: control group (CG), left brain-damaged patients not affected by visuo-spatial neglect (LN−), right brain-damaged patients without any sign of visuo-spatial neglect (RN−), and right brain-damaged patients affected by visuo-spatial neglect (RN+).** The vertical lines show the standard error of the mean (SEM) for each frequency (0.25, 2.0, and 4.0 cpd).

The group × frequency interaction was statistically significant [*F*_(3, 36)_ = 15.49, *p* < 0.01, η^2^ = 0.42]. The simple main effects were subsequently analyzed by three separate One-Way ANOVAs (Figure 2).

The comparisons of group (C, RN+, RN−, LN−) as an independent variable consistently revealed differences between groups [*F*_(3, 36)_ = 21.06, *p* < 0.01] for low spatial frequencies. The Duncan *post-hoc* test showed that the LN− (*p* < 0.001), RN− (*p* < 0.001), and RN+ (*p* < 0.002) groups needed more contrast to detect low spatial frequencies compared with the CG. The LN− group had lower sensitivity than the right RN+ (*p* < 0.002) and RN− (*p* < 0.001) groups.

In the analysis of high spatial frequencies, with group (C, RN+, RN−, and LN−) as the independent variable, differences were found between groups [*F*_(3, 36)_ = 71.82, *p* < 0.03]. The Duncan *post-hoc* test showed that all of the participants with lesions after stroke on the right side (RN− and RN+ groups) or left side (LN− group) had lower contrast sensitivity to detect high spatial frequencies compared with the CG (*p* < 0.01). The RN− (*p* < 0.05) and RN+ (*p* < 0.05) groups had lower contrast sensitivity compared with the LN− group (*p* < 0.01). Among the four groups, the RN+ group had the lowest sensitivity to high frequencies (*p* < 0.05).

Comparisons of medium spatial frequencies revealed a significant difference between groups [*F*_(3, 36)_ = 13.51, *p* < 0.01]. Differences were found between patients in the CG and the three groups who suffered strokes (*p* < 0.01; Duncan *post-hoc* test). No significant difference was found between right (RN− and RN+ groups) and left (LN− group) hemisphere lesions (*p* > 0.05).

## Discussion

The present study analyzed the importance of the right and LHs in detecting spatial frequencies in patients with unilateral brain lesions. The data demonstrated larger impairment in detecting high frequencies in patients with lesions in the LH (LN− group) and low frequencies in patients with lesions in the RH (RN− and RN+ groups). This suggests a double-dissociation between frequency and affected hemisphere (i.e., an advantage in high-frequency discrimination in the LN− group and advantage in low-frequency discrimination in the RN groups). However, the detection of medium frequencies was similar within groups, regardless of the affected hemisphere, and was only inferior compared with the CG.

The present results support the hypothesis about contrast sensitivity and hemisphere asymmetry that was initially proposed by Sergent (1982) to explain the input effects on visual hemifield asymmetry. Therefore, visual field and spatial frequency are related to hemispheric specialization in the processing of high and low spatial frequencies. Each hemisphere plays an essential role in perceptive stimulus detection (Mecacci, 1993).

Similar results were found by Kitterle and Selig (1991), who tested sensitivity in the detection of 1, 2, 4, 8, and 12 cpd presented to the left and right visual hemifield. The data obtained by these researchers suggested that several specific channels process each spatial frequency band.

Association patterns that are different from those described herein were found by other studies of frequency discrimination, which failed to find an interaction between hemisphere and spatial frequency (Ivry and Robertson, 1998; Niemeier et al., 2007). A failure to control changes in luminosity that follows stimulus presentation can interfere with spatial frequency processing. Other studies indicated that frequency detection tasks may produce asymmetric results under stimulation conditions with supraliminal contrast levels. According to these data, such conditions facilitate high-frequency discrimination (Grabowska et al., 1992; Proverbio et al., 1997). When the task involves decision making with regard to complex stimuli (e.g., face detection), the interaction between hemisphere and spatial frequency becomes more apparent (Kitterle et al., 1990).

Importantly, the task adopted in the present study did not involve the manipulation of perceptive factors that are able to induce superimposed asymmetry. Although specific variables, such as luminance, spatial frequency, eccentricity, and stimulus duration, were physically controlled, the participants focused their attention on detecting the target stimulus as opposed to the neutral stimulus, with variations only in the applied contrast. Additionally, the task demanded fast and precise discrimination between stimuli with the same luminance and directing attention to the center of the screen, compelling the subjects to use that information and promoting stimulus lateralization.

Although such neural lateralization has been primarily characterized by studies of perceptual and cognitive processes, behavioral and neuroimaging research has raised the possibility that the right and LHs play different roles in visual hemifields that are contralateral and ipsilateral to the lesion (Alves et al., 2008; Zhongming et al., 2009; Obregón and Shillcock, 2012). The present study did not find any interaction between these factors, which may be a limitation in the analysis and interpretation of the data. For future studies, further empirical research to test these patients with briefly presented lateralized stimuli seems to be warranted. Complementing brain damage and neuroimaging studies, the technique to divide visual fields has often been used in the investigation of brain asymmetry. The suitability of this method is based on the anatomical arrangement of the visual system, in which the temporal hemiretina sends information to the ipsilateral visual cortex, and the nasal hemiretina sends information to the contralateral visual cortex. Through the presentation of lateralized stimuli, analyzing differences in response times and judgment precision based on the visual field in which the stimuli are presented would have been possible.

The present study showed that patients in the RN+ group had lower sensitivity than patients in the RN− group for low and high frequencies. All of the participants in the groups with lesions in the RH (RN+ and RN− groups) were only better detecting low spatial frequencies, in contrast to the LN− group. Therefore, although the diminished sensitivity to high-frequency contrast was not attributable to the presence of visuo-spatial neglect, this condition negatively modifies spatial frequency perception. Evidence of basic visual processing deficiencies in individuals with visuo-spatial neglect has also been confirmed by electrophysiological studies (Doricchi et al., 1996). Visual-evoked potentials (VEPs) for contralateral stimuli are associated with a longer latency than VEPs for ipsilateral stimuli in these patients (Spinelli et al., 1996). This delay is typically associated with the presence of visuo-spatial neglect, and the deficit is curiously modulated by contrast and more acute with low-contrast stimuli.

In reference perceptual biases, we suggest the involvement of top-down and bottom-up processing in this study. Contrast sensitivity is a measure that evaluates the spatial processing bands of sensory channels. Bottom-up processing was involved in the present study. However, such participation cannot be regarded as exclusive because the lesions were in areas that are not principally visual but nonetheless alter visual contrast sensitivity.

A considerable debate has emerged over the past several years about the extent to which selection is controlled voluntarily (i.e., top-down processing) or automatically by the properties of the stimulus features in the environment (i.e., bottom-up processing). Based on the present results, we hypothesize that initially (i.e., the moment at which light hits the retina), visual selection is completely driven by the properties of the stimulus field. Only later does visual processing proceed in a top-down manner (Theeuwes, 2010). Importantly, top-down and bottom-up processing represent overlapping organizational principles rather than dichotomous constructs. In most situations, top-down and bottom-up processing interacts to optimize visual performance (Sarter et al., 2001).

A non-mutually exclusive alternative to the interpretation of the present results is that because attention is known to modify perception, attentional bias may also provoke perceptive bias (Niemeier et al., 2008). Angelelli et al. (1998) suggested that sensitivity loss may result from changes in the top-down attentional modulation of higher-order lesioned areas (e.g., parietal or parieto-temporal lobes) in visual cortices. This evidence provides further support for the hypothesis that there are at least two distinct regions in the human posterior parietal lobe (i.e., superior and inferior) that have quite different aspects of visuospatial processing. According to these authors, lesions of this inferior region may interfere in a lateralized way with the patient's ability related to spatial representations and hence cause visuo-spatial neglect (Angelelli et al., 1998).

Studies of spatial frequency detection suggest that specialized cortical mechanisms participate in the selection of information for high and low spatial frequencies (Martínez et al., 2001; Han et al., 2002). However, such processes appear to be related to specific neural operations, depending on the affected area and type of task. Therefore, electrophysiological and neuroimaging studies of different areas and task demands could contribute to the understanding of information processing mechanisms that involve brain lateralization.

In summary, the present study supported the hypothesis that contrast sensitivity is distributed bilaterally, with greater participation by the RH for low-frequency processing and by the LH for high-frequency processing. Although hemineglect can reduce contrast sensitivity, it does not appear to play a major role in the detection of spatial frequencies. This is the same pattern of presentation that other studies found correlated to perception, suggesting that spatial frequency detection involves specific neural substrates of spatial processing.

### Conflict of interest statement

The authors declare that the research was conducted in the absence of any commercial or financial relationships that could be construed as a potential conflict of interest.
